# Post-Dialysis Syndrome: A Narrative Review

**DOI:** 10.34067/KID.0000001062

**Published:** 2025-11-10

**Authors:** Gabriela Chacon-Palma, Maria-Eleni Roumelioti, Sarah Erickson, Jennifer L. Steel, Manisha Jhamb, Mark L. Unruh

**Affiliations:** 1 Division of Nephrology, Department of Internal Medicine, University of New Mexico School of Medicine, Albuquerque, New Mexico; 2 Department of Psychology, University of New Mexico, Albuquerque, New Mexico; 3 Department of Surgery, University of Pittsburgh School of Medicine, Pittsburgh, Pennsylvania; 4 Department of Psychiatry, University of Pittsburgh School of Medicine, Pittsburgh, Pennsylvania; 5 Department of Psychology, University of Pittsburgh School of Medicine, Pittsburgh, Pennsylvania; 6 Renal-Electrolyte Division, Department of Medicine, University of Pittsburgh School of Medicine, Pittsburgh, Pennsylvania

**Keywords:** chronic dialysis, chronic hemodialysis, CKD, clinical nephrology, dialysis, ESKD, hemodialysis, kidney failure, renal dialysis

## Abstract

Use of the term post-dialysis syndrome (PDS) was recently recommended to refer to the exacerbation of a debilitating, under-recognized cluster of physical, cognitive, psychological, and behavioral symptoms commonly affecting patients on maintenance dialysis after treatment sessions. This narrative review synthesizes existing and emerging literature to provide an overview of the characterization, pathophysiology, measurement, and management of PDS. Commonly referred to as post-dialysis fatigue, the term PDS more accurately captures the multidimensional nature and true burden of the condition and may minimize patient stigma. Its pathophysiology is multifactorial, and the evidence suggests that inflammation, nutritional status, and dialysis-related, clinical, sociodemographic, and psychiatric factors are significant contributors. Although a number of measurement tools and interventions targeting individual symptoms have been studied, there are no established methods or guidelines to define, diagnose, and manage PDS in its entirety. Existing quantitative instruments include time to recovery from dialysis, self-report scales, and ecological momentary assessments, but they differ in their ability to capture the heterogeneity of and variations in PDS symptoms and in psychometric properties. Qualitative and mixed-methods approaches may provide more in-depth information on the scope and effect of PDS and can be useful for future development of better measurement tools. Potential interventions for PDS include adjustment of dialysis prescription, exercise and physical activity, and cognitive behavioral therapy. Studies examining these interventions have focused on a narrow range of post-dialysis symptoms, however, and given the lack of PDS management guidelines, patients strongly advocate for further research into treatments. In the interim, we recommend a pragmatic approach to address PDS in dialysis patients involving: collection of a detailed medical history to understand the range, severity, and impact of post-dialysis symptoms; use of patient-reported outcome measures to quantify symptoms and treatment effects; and engagement in shared decision-making with patients when selecting among interventions.

## Introduction

Patients on long-term hemodialysis experience a large burden of symptoms that negatively impact quality of life (QOL).^1,2^ Use of the term post-dialysis syndrome (PDS) was recently recommended to refer to the exacerbation of a cluster of physical, cognitive, psychological, and behavioral symptoms that may occur after dialysis affecting daily functioning.^3^ Its high prevalence and profound, multidimensional effect on patients and families make PDS a critical issue for individuals receiving maintenance hemodialysis (MHD). Of patients on MHD, ≥50% experience debilitating fatigue from treatment and/or post-dialysis decreased stamina and energy levels,^4^ suggesting the true prevalence of PDS—encompassing a myriad of other potential manifestations—is likely higher. For some, this results in severe impairment that limits life participation and leads to challenges with relationships, independence, feelings of purpose, and employment.^4^ Despite this, PDS remains poorly defined,^5^ and there are limited reliable methods for its diagnosis and management.^6^ This narrative review synthesizes existing and emerging literature to provide an overview of the newly introduced term PDS, encompassing post-dialysis fatigue (PDF) and the range of associated symptoms commonly affecting patients on maintenance dialysis after treatment sessions.

## Literature Search Strategy

We used PubMed, which allows for searches across MEDLINE, PubMed Central, and Bookshelf, to select randomized controlled trials (RCTs), observational studies, systematic and narrative reviews, meta-analyses, and other published works (*e.g*., perspectives) relevant to PDS. In addition, we searched the gray literature using Google Scholar for other PDS-related manuscripts, such as PhD theses. Search terms and keywords included PDS, PDF, fatigue, ESKD, end-stage renal disease, (post-) dialysis recovery time (DRT), and (hemo) dialysis. Additional search terms were explored, including pathophysiology, inflammation, oxidative stress, mineral metabolism, nutrition, amino acids (AA), patient-reported outcome measures (PROM), ecological momentary assessment (EMA), quantitative measurement, psychometric properties, qualitative measurement, treatment, management, dialysis prescription, exercise, physical activity, cognitive behavioral therapy (CBT), and carnitine. We chose a narrative design to best explore the broad and relatively ill-defined nature of PDS. As such, study selection was primarily informed by the literature available on PDS itself as well as on PDF and fatigue in dialysis. Nonetheless, we also provide evidence on other components of PDS, such as physical and mental manifestations.

## Defining and Characterizing PDS

**Figure 1:**
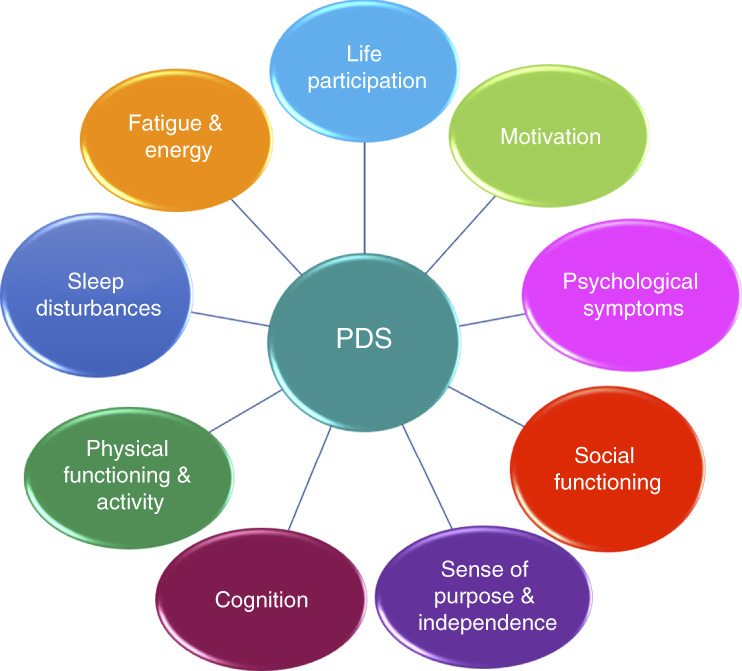
**Relationship of PDS to health-related QOL and functioning.** PDS, post-dialysis syndrome; QOL, quality of life.

While there is no established definition of PDS, there is consensus among patients and clinicians that an accurate characterization must capture the ways in which PDS is beyond a feeling of tiredness; it is a whole-body experience with cognitive, psychological, and physical components.^5,7^ Some patients view it as an overwhelming exhaustion that leaves them with limited intradialytic well-being and traps them in an endless cycle of weakness and recovery periods.^8–11^ Others note the negative effect of PDS on life participation, an inability to engage in joyful interests/hobbies, and the need to adapt their lifestyle to manage diminished energy (Figure 1).^9,12–14^ In a 2025 study, patients on MHD reported significantly lower post-dialysis positive mood and alert cognition and higher post-dialysis negative mood and sleepiness/fatigue compared with similar time points on nondialysis days.^15^

Participants of the 2023 A Scientific Workshop on PDF, hosted by the National Institute of Diabetes and Digestive and Kidney Diseases, proposed renaming PDF to more accurately capture its multidimensional nature and to minimize patient stigma from limited understanding of its true burden.^5^ The proposed term PDS can change misconceptions by identifying this phenomenon not as a single symptom, but rather a heterogeneous cluster of symptoms affecting multiple aspects of health and QOL. Future research with a larger dialysis cohort could gather additional insight on patient perspectives regarding a terminology change and experiences with PDS versus PDF.

The need for a clear and widely accepted definition of PDS remains, however. A comprehensive characterization will ideally encapsulate all factors associated with this pervasive condition, including fatigue; psychological symptoms; sleep disturbances; and impaired social functioning, cognition, and physical activity (Figure 1).^15–18^ Some advocate for establishing a set of criteria to accurately identify PDS.^5^ This can be achieved by leveraging the strengths of quantitative and qualitative methods (see Measurement section), as was done when constructing a case definition for Gulf War syndrome through a systematic approach using interviews and surveys.^5,19^ More research on patients' experiences and perspectives is needed to better understand this phenomenon as a syndrome, thereby facilitating the development of improved diagnostic tools.

## Pathophysiology

**Figure 2:**
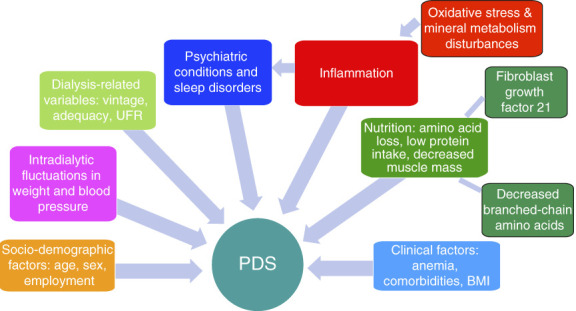
**Pathophysiologic mechanisms implicated in PDS.** BMI, body mass index; UFR, ultrafiltration rate.

The pathogenesis of PDS is multifactorial and the underlying mechanisms are not fully understood (Figure 2). Factors contributing to PDS include inflammation^20,21^; nutritional status^22^; and dialysis-related,^16,23–25^ clinical,^25–27^ sociodemographic,^16,25,28^ and psychiatric variables.^16,29,30^

Inflammation is prevalent among dialysis patients^31^ and plays a role in PDS by promoting fatigue, sleep disorders, depression, and cognitive impairment.^32^ The proinflammatory cytokines IL-6^21^ and tumor necrosis factor-α^20^ have been associated with fatigue in chronic hemodialysis^21^ and PDF,^20^ respectively. Oxidative stress, an inflammation mediator, is augmented by hemodialysis^33,34^ and has been implicated in the pathophysiology of hemodialysis-related fatigue^35^ and uremic myopathy—characterized by fatigue, muscle weakness/wasting, and exercise intolerance.^36^ Mineral metabolism disturbances, common in hemodialysis,^37^ also promote inflammation^38,39^ and contribute to PDS. For example, a case-control study showed a significant association between higher postdialysis serum calcium and PDF.^40^

Nutritional status is another contributing factor, affecting PDS symptoms such as PDF and sleep quality.^22^ Hemodialysis is a protein-catabolic process with AA loss and a net negative protein balance in the body and skeletal muscle compartment.^41^ In one study, patients on MHD (versus controls) lost seven times more AAs daily and 6.7% versus 0.7% of their total protein intake.^42^ In addition, higher protein intake was associated with lower odds of severe fatigue, affecting physical activity, concentration, and/or motivation.^42^ Possible mechanisms include maintenance of muscle mass/strength and diminished protein-energy wasting, which affect physical functioning and fatigue.^42^ Another investigation showed that plasma fibroblast growth factor 21 levels increased during intradialytic periods and were associated with higher odds of low protein intake, lower muscle mass, and more fatigue in MHD.^43^ Finally, postdialysis branched-chain AA (BCAA) plasma levels have been shown to be significantly lower compared with predialysis levels and were negatively associated with fatigue on hemodialysis days.^44^ This may be due to BCAAs' role in neurotransmitter synthesis, *e.g*., brain 5-hydroxytreptamine regulating arousal, sleep, and mood,^45,46^ and the fact that BCAAs comprise 35% of essential AAs in muscle proteins,^46^ potentially resulting in decreased muscle mass/strength when depleted.^44^

Dialysis-related factors are implicated in the pathogenesis of PDS. Longer DRT,^16^ fatigue,^24^ and PDF intensity, duration, and sum score (intensity, duration, frequency)^23^ in MHD patients have been associated with dialytic vintage. Studies on MHD reported that DRT^16^ and PDF^25^ were positively associated with intradialytic weight loss^16^ and interdialytic weight gain,^25^ respectively. There is also evidence for a relationship between PDF and inadequate dialysis.^25^ In addition, investigations on chronic hemodialysis reported a negative association between ultrafiltration rate and DRT^47^ and PDF intensity and sum scores.^23^ However, a 2024 study found no differences in intradialytic symptoms or DRT between different UFRs.^48^

Sociodemographic, clinical, and psychiatric factors also contribute to PDS. Studies have shown positive associations between age and DRT^16^ and PDF^25,28^ and between shorter DRT and male sex and full-time employment.^16^ PDF has been associated with lower hemoglobin^25^ and depression,^29^ and DRT has been associated with psychiatric disorders^16^ and depression.^30^ Another investigation found a positive relationship between PDF and sleep disorders and comorbidities.^27^ Possible mechanisms include increased stress caused by multiple pathologies and medication side effects and interactions.^27^ Finally, DRT was found to increase as body mass index increases, potentially because of increased proinflammatory cytokine levels in patients with higher BMIs.^26^

## Measurement

There are no guidelines or consensus on how to measure PDS.^49,50^ Various approaches and instruments have been used in clinical and research settings, but they differ in psychometric properties and ability to capture the heterogeneity of PDS symptoms (Table 1).

**Table 1:** Measurement of postdialysis syndrome

Assessments and Approaches	Studies	Strengths	Limitations
**Quantitative measurements**			
Time to recovery from dialysis	- Lindsay *et al.* 2006^51^	- Demonstrated to be valid	- Does not directly assess range of PDS symptoms
*Single-item measure*:	- Rayner *et al.* 2014^16^	- Ease of interpretation	- Does not assess effect of PDS on different life domains
- “*How long does it take you to recover from a dialysis session?*”^16,51^	- Garg *et al.* 2017^52^	- Used in multiple studies	- Does not reflect day-to-day or diurnal variations
- *Patient responses recorded in min/h or as a categorical choice*	- Davenport *et al.* 2018^30^		- Subject to recall bias
	- Alvarez *et al.* 2020^53^		
	- Elsayed *et al.* 2022^54^		
General fatigue self-report scales			
- *SF-36*^55,56^*: vitality subscale*^57^	- Lee at al. 1991^63^	- Some scales, *e.g*., SF-36, have been widely used across numerous studies and clinical/research settings	- Most measure fatigue only and do not assess associated PDS symptoms
- *22-Item revised-piper fatigue scale*^57–59^	- McHorney *et al.* 1993^55^	- The brief fatigue inventory and chalder fatigue questionnaire measure some of the impact-related dimensions of fatigue in dialysis	- Prone to recall bias
- *20-Item multidimensional fatigue inventory*^60,61^	- Smets *et al.* 1995^60^	- Scales vary in length and participant burden, ease of comprehension, and psychometric properties	- Most assess fatigue on average over a certain time duration (usually 1 wk)
- *13-Item functional assessment of chronic illness therapy fatigue*^62^	- Mingardi *et al.* 1999^56^		- Scales vary in length and participant burden, ease of comprehension, and psychometric properties
- *18-Item visual analog scale for fatigue*^49,63^	- Dagnelie *et al.* 2006^58^		
- *Seven-Item PROMIS fatigue-short form and computer-adaptive test*^64,65^	- Ameringer *et al.* 2016^64^		
- *Ten-Item brief fatigue inventory*^66^	- Chao *et al.* 2016^62^		
- *11-item chalder fatigue questionnaire*^67^	- Picariello *et al.* 2016^67^		
	- Chilcot *et al.* 2017^61^		
	- Ju *et al.* 2018^57^		
	- Ju *et al.* 2020^59^		
	- Kodama *et al.* 2020^49^		
	- Dano *et al.* 2023^65^		
	- Debnath *et al.* 2023^66^		
SONG-HD fatigue^59^			
- *Three-item questionnaire*	- Ju *et al.* 2020^59^	- Shown to be valid	- Does not capture other associated PDS symptoms
		- Low patient burden	- Subject to recall bias
		- Provides information that is meaningful and relevant to patients, caregivers, and clinicians	
		- Assesses various dimensions of fatigue and effect on daily activities	
EMAs			
*Item number varies*:	- Shiffman *et al.* 2008^72^	- Reflect day-to-day or diurnal changes in symptoms and associated behavioral/social factors	- Subject to patient compliance
- *Repeated real-time measurements using computer-assisted telephone interviews*;^3^ *automated telephone- administered scales*;^15^ *diary entries*;^68^ * mobile applications*;^69,70^ *wearables, e.g*., *accelerometers*;^70^ and *smartwatches*^71^	- Brys *et al.* 2019^68^	- Data captured in patients' natural environments	- Frequency of assessments may impose greater patient burden
	- Brys *et al.* 2020^69^	- Wearables (*e.g*., accelerometers, smartwatches) can provide real-time, accurate data on physical activity	- May be subject to observer (Hawthorne) effect
	- Laborde *et al.* 2021^71^		- Reliance on technology or means of communication may be limiting for patients
	- Kallem *et al.* 2024^3^		
	- Tarca *et al.* 2024^70^		
	- Kallem *et al.* 2025^15^		
**Qualitative approaches**			
- One-on-one semistructured patient interviews during dialysis sessions, in private at treatment center, or at home^8,9,12,73,74^	- Heiwe *et al.* 2003^9^	- Ability to gather more in-depth information on perceptions of PDS, including its scope and effect	- Providing answers in real time to interviewer/host may affect patient responses
- Small-group discussions part of focus or nominal groups hosted by health care professionals^75^	- Horigan *et al.* 2013^12^	- Helpful for future development of measurement tools capable of capturing all PDS dimensions and formulation of a case definition	- Greater patient burden
	- Urquhart-secord *et al.* 2016^75^	- May incorporate stakeholder engagement to better address feasibility and acceptability of new tools	- Small-group discussions not optimal for openness and voicing experiences for all patients
	- Cox *et al.* 2017^8^		- Do not assess population-level prevalence or effect
	- Rezaei *et al.* 2018^73^		
	- Flythe *et al.* 2019^74^		
**Mixed-methods approach**			
- Patients provide comments supplementing answers to scales^76^	- Ju *et al.* 2020^76^	- Comments and interviews provide more details and depth to answers selected on scales	- Subject to recall bias
- Explanatory sequential study design: baseline and intervention phase questionnaires followed by postintervention interviews^77^	- Farragher *et al.* 2020^77^	- Comments and interviews can collect data on PDS dimensions not captured by scales	- Greater patient burden when combining approaches
		- Helpful for future development of measurement tools capable of capturing all PDS dimensions and formulation of a case definition	- May not capture all PDS symptoms depending on details provided beyond questions directly asked on scales
		- Depending on the scale(s), can benefit from ease of comprehension, patient burden, psychometric properties, and prior use in various studies/settings	

EMA, ecological momentary assessments; PDS, post-dialysis syndrome; PROMIS, patient-reported outcome measurement information system; SF-36, 36-item short form; SONG-HD, standardized outcomes in nephrology-hemodialysis.

PDS can be measured as time to recovery from dialysis.^16,51^ Patients are asked, “How long does it take you to recover from a dialysis session?” and responses are recorded in minutes/hours or as a categorical choice. While time to recovery is a validated question with ease of interpretation^51^ and has been used in multiple studies,^30,52–54^ it does not directly assess the severity and breadth of symptoms comprising PDS.

Most existing quantitative instruments measure fatigue only or some, but not all, PDS symptoms. The vitality subscale of the 36-item short form (SF-36)^55,56^ is a reliable, widely used tool in clinical and research settings to measure fatigue in hemodialysis.^57^ Other fatigue scales used in dialysis include the Revised-Piper Fatigue Scale,^57–59^ Multidimensional Fatigue Inventory,^60,61^ Functional Assessment of Chronic Illness Therapy Fatigue,^62^ Visual Analog Scale for Fatigue,^49,63^ and Patient-Reported Outcome Measurement Information System Fatigue-Short Form and Computer-Adaptive Test.^64,65^ Examples of instruments used in dialysis measuring additional PDS dimensions include other SF-36 subscales assessing physical and social functioning and mental health,^55,56^ the Beck Depression Inventory,^78^ the Pittsburg Sleep Quality Index,^79^ and the Index of Activities of Daily Living.^80^ The Kidney Disease QOL 36-item (KDQOL-36) survey,^81^ which includes Short Form-12,^82^ is commonly used in clinical settings and measures general physical and emotional health; specific symptoms accompanying PDS (*e.g*., pain, dizziness, shortness of breath); and energy levels and feeling washed out or drained.^81^ These assessments vary in length, ease of comprehension, and burden to respondents. To mitigate some of the short-comings of existing fatigue scales, the Standardized Outcomes in Nephrology-Hemodialysis initiative developed a PROM for fatigue designed to be valid, brief, and able to produce critical, meaningful information for patients and clinicians. Standardized Outcomes in Nephrology-Hemodialysis Fatigue is a three-item measure that assesses tiredness, energy levels, and the effect of fatigue on daily activities.^59^

**Figure 3:**
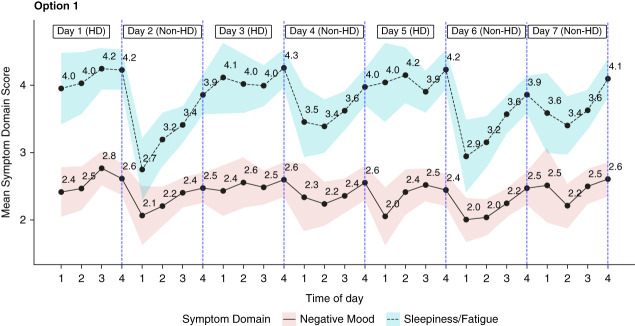
**Mean postdialysis and nondialysis negative mood and sleepiness/fatigue scores at all time points.** For negative mood and sleepiness/fatigue, higher scores are worse. Means plotted include all EMA data that patients provided after their hemodialysis sessions on hemodialysis days as well as all EMA data that patients provided at the same time points on nonhemodialysis days. Error bands depict the 95% CI around the estimated mean at each time point. Time of day 1=morning, 2=early afternoon, 3=late afternoon, and 4=evening. For patients on a Monday-Wednesday-Friday dialysis schedule, day 1=Monday; for patients on a Tuesday-Thursday-Saturday dialysis schedule, day 1=Tuesday. Average Negative Mood scores by time point: 1=2.42, 2=2.52, 3=2.53, and 4=2.55. Average sleepiness/fatigue scores by time point: 1=3.60, 2=3.78, 3=3.80, and 4=4.09. Reproduced from Kallem *et al*.^15^ CI, confidence interval; EMA, ecological momentary assessment; HD, hemodialysis.

Most conventional instruments measure symptoms retrospectively, however, making them prone to recall bias and unable to reflect variations within the period specified by questionnaires.^83^ By contrast, EMAs involve repeated measurements of symptoms and behavioral and social factors in real time and in patients' natural environments, allowing them to capture day-to-day or diurnal changes.^72^ EMAs can be conducted using PDS-related questionnaires completed multiple times per day through computer-assisted telephone interviews^3^ and mobile applications.^69,70^ A recent study conducted EMAs at four daily time points *via* automated telephone-administered scales to compare postdialysis and nondialysis day symptoms.^15^ Figure 3 (reproduced from Kallem *et al*.^15^) depicts that negative mood and sleepiness/fatigue scores were significantly higher postdialysis than on nondialysis days.^15^ EMAs may also be completed through PDS-oriented fatigue diary entries at predetermined times and days.^68^ In addition, wearables, *e.g*., wrist-worn accelerometers^70^ and smartwatches,^71^ provide real-time information, such as physical activity, and complement other data collection methods.

The use of qualitative and mixed-methods approaches can provide more in-depth information on perceptions of PDS, including its scope and extent of impact, allowing for the development of measurement tools that capture all its dimensions and for formulation of a case definition. One common qualitative method is conducting one-on-one semistructured interviews with patients during treatment sessions, in private rooms at their dialysis center, or at home.^8,9,12,73,74^ For example, Symptom Monitoring in Renal Replacement Therapy-Hemodialysis is a PROM developed through concept elicitation interviews and refined through cognitive debriefing interviews, and its effectiveness is currently being evaluated in the Symptom Monitoring in Renal Replacement Therapy-Hemodialysis trial.^74,84^ Another example is facilitating small-group discussions as part of focus/nominal groups hosted by health care professionals.^75^ Patients are asked open-ended questions regarding their perspective on PDS and its effect on physical functioning, sleep, psychological factors, interpersonal relationships, employment, and other symptoms or experiences. Qualitative approaches can also help address the feasibility and acceptability of novel assessment tools through stakeholder engagement. A mixed-methods approach, wherein patients provide comments supplementing their answers to scales, may provide additional insight.^76^

Finally, it is critical to establish reliable, valid instruments to not only accurately measure the severity and breadth of PDS symptoms but also to assess their impact on different life domains. A systematic review on PROMs for fatigue in hemodialysis patients identified 11 content dimensions: life participation, motivation, negative emotions, ability to concentrate, ability to think clearly, memory, verbal abilities, energy level, PDF, tiredness, and limb/muscle weakness.^57^ Of these, the first seven dimensions capture the effect of fatigue on mental and physical functioning, but only life participation was frequently included in fatigue measures (33%).^57^ For example, the Brief Fatigue Inventory^66^ and Chalder Fatigue Questionnaire^67^ assess two and five of the seven impact-related dimensions, respectively. However, they lack most psychometric properties identified by the COnsensus-Based Standards for the selection of health Measurement INstruments-Core Outcomes Measures in Effectiveness Trials guidelines.^85^ The effect of PDS can also be described using qualitative or mixed-methods approaches. This is illustrated by the mixed-methods explanatory sequential design used in a study of patients on chronic dialysis, consisting of baseline and intervention-phase questionnaires on fatigue and life participation followed by postintervention interviews.^77^

More research is needed to establish instruments that accurately gather data on the variety of measurement and content dimensions of PDS, including those measuring its effect on different life domains and that meet the psychometric properties recommended by COnsensus-Based Standards for the selection of health Measurement INstruments-Core Outcomes Measures in Effectiveness Trials. Moreover, it is important that assessment tools are tailored to the unique challenges of the dialysis population, have clinical applicability, and have the capacity to capture day-to-day or diurnal variations. Measurement instruments meeting these characteristics are essential in the development of effective PDS treatments.

## Management

Patients on hemodialysis strongly express the need for more research on the development and implementation of treatments for PDS.^5^ This section explores the evidence behind various interventions (Table 2), including adjustment of dialysis prescription, exercise and physical activity, and CBT, that reportedly improve ≥1 PDS symptoms. The literature presented primarily focuses on PDF, DRT, and fatigue in MHD.

**Table 2:** Management of post-dialysis syndrome

Study	Treatment/Variable	Study design	Findings
**Dialysis prescription**			
Faioli *et al.* 2024^27^	Number of weekly dialysis sessions	Observational	Number of weekly sessions (≥3 versus ≤2) was positively correlated with PDF score
		- 250 patients with CKD on hemodialysis	
		- Cross-sectional survey	
Garg *et al.* 2017^52^	Frequent hemodialysis (six times a week)	Parallel-arm RCTs	Both trials: After 1 yr, decreased postsession and total weekly recovery times when switched to frequent hemodialysis
		- Daily trial: 245 patients on conventional (three times) maintenance hemodialysis assigned to frequent or conventional in-center hemodialysis	
		- Nocturnal trial: 87 patients on conventional maintenance hemodialysis assigned to frequent nocturnal or conventional hemodialysis at home	
		- Both trials: 12-mo follow-up	
Jaber *et al.* 2010^86^	Daily hemodialysis (six times a week)	Observational	Daily hemodialysis was significantly associated with lower depression scores (over last 2 wk from assessment) and shorter post-DRT over 12 mo
		- 128 adults with ESKD requiring dialysis initiated on daily home hemodialysis	
		- Minimum 12-mon follow-up	
Davenport *et al.* 2019^87^	Incremental hemodialysis	Observational	Incremental hemodialysis (versus conventional hemodialysis) was associated with shorter post-DRT
		- 709 total patients on chronic hemodialysis across 5 dialysis centers	
		- One center: 254 adults on incremental hemodialysis (reduction of dialysis session length based on RKF)	
		- Four centers: 455 adults on conventional hemodialysis (∼4-hour sessions, 3×/wk)	
		- Minimum 12-mo follow-up	
Rayner *et al.* 2014^16^	Dialysate sodium concentration	Observational	Lower dialysate sodium concentration was associated with longer DRT.
		- 6040 patients on maintenance hemodialysis in phase 4 of DOPPS	
		- Median 12-mo follow-up	
Elsayed *et al.* 2022^54^	Dialysate sodium	Observational	Dialysate sodium was inversely correlated with DRT
		- 191 adults on regular, long-term hemodialysis	
		- Cross-sectional survey	
Levin *et al.* 1996^88^	RHSD	Crossover RCT	RHSD improved postdialysis hangover in 100% of participants initially reporting it. 56% of participants reported improved overall energy for recreational activities on RHSD
		- 16 patients on chronic hemodialysis given RHSD for a 2-wk run-in period followed by RHSD and conventional hemodialysis for 3 wk each in randomized sequence	
		- Total 8 wk duration	
Bossola *et al.* 2020^23^	UFR	Observational	PDF intensity and PDF sum score (duration, intensity, frequency) were negatively associated with UFR
		- 271 patients on chronic, conventional hemodialysis	
		- Each patient interviewed during one regularly scheduled treatment	
Bossola *et al.* 2019^47^	UFR	Observational	UFR was inversely associated with post-DRT
		- 210 patients on chronic hemodialysis	
		- Mean of last five UFR values collected at time of inclusion	
		- Recovery time recorded after hemodialysis session	
Sajadi *et al.* 2016^89^	Cool dialysate	Crossover RCT	Cool dialysate resulted in a significant improvement in predialysis versus postdialysis fatigue scores: Total and behavioral, emotional, sensory, and cognitive dimensions of the PFS
		- 46 adults with fatigue on chronic hemodialysis received three hemodialysis sessions with dialysate at 37°C or 35.5°C in first wk followed by another week on other dialysate temperature	
		- Total 2 wk duration	
		- PFS completed at baseline and after each three sessions of hemodialysis	
Karkar *et al.* 2015^90^	High-efficiency postdilution online HDF	Parallel-arm RCT	High-efficiency postdilution online HDF (versus high-flux hemodialysis) improved PDF; QOL, including social, physical, and professional activities; and mood
		- 72 patients on chronic, regular low-flux hemodialysis randomized to high-flux hemodialysis or high-efficiency postdilution online HDF	
		- 24 mo follow-up	
Smith *et al.* 2017^91^	Online postdilution HDF	Crossover RCT	There was no difference in post-treatment recovery time between hemodialysis and HDF There was no difference in physical and mental health composite scores on kidney disease quality of life-short form between hemodialysis and HDF after 8 wk of each treatment
		- 100 patients on chronic hemodialysis randomized to 8 wk of hemodialysis followed immediately by 8 wk of online postdilution HDF or *vice versa*	
		- Recovery time assessed for prior session on arrival for each treatment	
Bossola *et al.* 2023^92^	Online HDF	Observational	Recovery time after dialysis and PDF did not significantly differ between bicarbonate hemodialysis and online HDF
		- 252 patients on chronic bicarbonate hemodialysis	
		- 83 patients on chronic online HDF	
		- Cross-sectional survey	
Bolton *et al.* 2021^93^	HD×	Observational	Patients switched to HD× reported significantly shorter post-DRT and lower severity of general fatigue/lack of energy (over past wk from assessment) that were sustained at 12 mo
		- 58 patients initially on conventional, high-flux hemodialysis transitioned to HD× (using medium cut-off membrane) by dialysis unit renal team after implementation of PROM assessments	
		- Up to a 12 mo observation period with baseline and quarterly assessments administered while on dialysis, usually at a mid-week session	
Nakayama *et al.* 2025^94^	Hemodialysis employing molecular hydrogen-enriched dialysis solution rendered by water electrolysis (E-HD)	Observational	At 12 mo after switch to E-HD, significantly lower general fatigue scores on hemodialysis and hemodialysis-free days among patients with persistent fatigue (on both hemodialysis and hemodialysis-free days) or with fatigue only on hemodialysis days when compared with baseline measurements
		- 81 patients on standard dialysis switched to 12 mo of E-HD	
		-Fatigue assessments performed during standard hemodialysis period within 2 wk before start of E-HD and at months 1, 3, 6, and 12 after E-HD commencement	
		-Total 12 mo observation period	
**Exercise and physical activity**			
Zhou *et al.* 2023^95^	Intradialytic exercise: Aerobic+resistance	Single-arm trial	Patients reported significant improvements across time in general fatigue (experienced lately from time of assessment), depression (mostly current symptoms at time of assessment), and health-related QOL
		- 75 patients on regular, maintenance hemodialysis completed 12 wk of three times a week, 30 min per hemodialysis session intradialytic exercise	
		- Data collection at baseline, immediately postintervention, and 3 mo postintervention	
Grigoriou *et al.* 2021^96^	Intradialytic exercise: Aerobic+resistance	Single-arm trial	After 9-mo intervention, participants reported feeling better after dialysis, lower PDF severity, shorter PDF duration, and improved cognitive function, SF-36 vitality subscale, depression (over past several days from time of assessment), and chronic fatigue scores.
		- 20 patients on chronic hemodialysis completed a 9-mo, three times a week, 60–80 min per hemodialysis session intradialytic exercise program	
		- Assessments before and after 9 mo of exercise intervention	
Samuel raj *et al.* 2023^97^	Intradialytic exercise: Flexibility+strengthening+endurance	Parallel-arm RCT	At end of 36 wk, intervention group had a clinically significant decrease in general fatigue from baseline. At 24 and 36 wk, there was a significant difference in general fatigue scores between experimental and control groups (in favor of intervention).
		- 30 patients with ESKD on outpatient hemodialysis completed study with randomization to a 36-wk, two times a week intradialytic exercise program or to no specified exercise (control)	
		- Fatigue assessments at 0, 12, 24, and 36 wk	
Lakshmi *et al.* 2024^98^	Intradialytic exercise: Aerobic	Nonrandomized interventional	Postdialysis fatigue scores improved in experimental versus control group
		-148 patients on chronic hemodialysis performed up to 8 wk of three times a week, 15 min per hemodialysis session intradialytic exercise	
		- 147 patients on chronic hemodialysis to follow routine care only (control)	
		- Fatigue assessed at end of the 8 wk immediately after final dialysis session	
Gordon *et al.* 2011^99^	Physical activity	Observational	PDF was inversely correlated with physical activity.
		- 58 patients on hemodialysis	
		- Physical activity assessed by self-report and accelerometry	
		- Cross-sectional survey	
Leme *et al.* 2020^100^	Physical activity/step counts	Observational	Chronic fatigue was negatively associated with physical activity (step counts) in the 24 h posthemodialysis period
		- Analysis of baseline data from 176 patients on chronic hemodialysis part of RCT	
		- Physical activity (step counts) measured by accelerometry	
		-Cross-sectional survey	
**Cognitive behavioral therapy**			
Mehrotra *et al.* 2019^101^	CBT	Parallel-arm RCT	At 12 wk, both groups had a clinically significant improvement in depression (over past 7 d from assessment), disability, energy/vitality subscale of SF-36, and anxiety (over last 2 wk from assessment) scores from baseline, but scores were modestly better with sertraline for the first three outcomes
		- 120 patients on maintenance hemodialysis with major depressive disorder or dysthymia randomized to ten CBT sessions, 60 min each, over 12 wk or to sertraline treatment	
		- Assessments at baseline and 6 and 12 wk	
Jhamb *et al.* 2023^102^	CBT and/or pharmacotherapy	Parallel-arm RCT	Intervention group (versus control) had statistically and clinically significant reductions in general fatigue and pain severity at 3 mo and sustained at 6 mo as well as statistically significant improvement in depression (over past 2 wk from assessment) at 3 mo
		- 160 adults on maintenance hemodialysis with clinically significant levels of fatigue, pain, and/or depression randomized to 12 weekly CBT sessions through telehealth and/or pharmacotherapy using a stepped collaborative approach or to six biweekly sessions of health education (control)	
		- 12-mo follow-up	
Picariello *et al.* 2021^103^	CBT for renal fatigue	Parallel-arm feasibility RCT	There were moderate to large treatment effects in favor of CBT intervention on fatigue severity (over last mo from assessment), fatigue-related functional impairment (current at time of assessment), depression (over past 2 wk from assessment), and anxiety (over past 2 wk from assessment
		- 24 patients on chronic hemodialysis with clinical levels of fatigue randomized to CBT aimed at fatigue specifically (self-management manual accompanied by 3–5 sessions) or to usual renal care (control)	
		- Assessments at baseline and at 3 mo postrandomization	

CBT, cognitive behavioral therapy; DOPPS, Dialysis Outcomes and Practice Patterns Study; DRT, dialysis recovery time; E-HD, hemodialysis employing molecular hydrogen-enriched dialysis solution rendered by water electrolysis; HDF, hemodiafiltration; HD×, expanded hemodialysis; PDF, post-dialysis fatigue; PFS, piper fatigue scale; PROM, patient-reported outcome measure; QOL, quality of life; RCT, randomized controlled trial; RHSD, ramped hypertonic sodium dialysis; RKF, residual kidney function; SF-36, 36-item short form survey; UFR, ultrafiltration rate.

### Dialysis Prescription

Targeted interventions on dialysis-related factors can mitigate some manifestations of PDS. Studies have demonstrated a correlation between the number of weekly dialysis sessions, PDF,^27^ and DRT.^52^ In two RCTs, patients undergoing conventional MHD (three times a week) who were switched to frequent hemodialysis (six times a week) reported decreased postsession and total weekly recovery times compared with those who remained on conventional hemodialysis.^52^ In another study, patients requiring dialysis transitioned to daily hemodialysis (six times a week) had significantly lower depression scores and shorter DRT over 12 months.^86^ Incremental hemodialysis—individualization of hemodialysis duration or frequency based on residual kidney function (RKF)—has been associated with lower PDF^104^ and shorter DRT.^87^ Incremental hemodialysis involves continuous measurement of RKF with the ultimate goal of preserving it. Accounting for RKF when tailoring hemodialysis prescriptions may allow for less intensive ultrafiltration and reduced session duration, potentially resulting in shorter DRT.^87^

Similar to previous studies,^16,88,105^ Elsayed *et al.*^54^ observed that DRT was inversely correlated with dialysate sodium in patients on MHD.^54^ In another study, intradialytic hypotensive episodes prolonged the median DRT by over three-fold in MHD.^26^ Cool dialysate, however, has been found to reduce the frequency of intradialytic hypotension^106^ and has been recommended as an intervention to reduce PDF in chronic hemodialysis patients, particularly those with severe fatigue.^89^ Use of online hemodiafiltration (HDF), which combines diffusion and convection to improve clearance of middle molecules, *e.g*., inflammatory cytokines, to reduce PDS is inconclusive. One RCT found that HDF versus high-flux hemodialysis improved various PDS domains: PDF; QOL, including social, physical, and professional activities; and mood.^90^ Other investigations found no difference in PDS symptoms among patients on hemodialysis versus HDF.^91,92^ In another study, patients on conventional hemodialysis transitioned to expanded hemodialysis, which uses a medium cut-off membrane to more effectively clear large middle molecules, reported significantly shorter post-DRT and lower severity of general fatigue/lack of energy that were sustained at 12 months after the switch.^93^ Finally, Nakayama *et al.* observed significantly lower general fatigue scores on hemodialysis and hemodialysis-free days in patients with persistent fatigue or with fatigue only on hemodialysis days after transitioning from conventional dialysis to hemodialysis employing molecular hydrogen-enriched solution.^94^

These interventions may be limited by patient preference, *e.g*., more intradialytic symptoms with cool dialysate and increased time spent in treatment on frequent dialysis regimens, and access to different dialysis modalities. In addition, many of these studies are observational and focus on a narrow range of PDS symptoms.

### Exercise and Physical Activity

Various studies have demonstrated the benefits of interdialytic/intradialytic exercise and physical activity in the dialysis population. In a recent trial, patients on MHD participating in a 12-week, thrice-weekly, intradialytic group exercise program reported significant improvements in general fatigue, depression, and health-related QOL.^95^ Similarly, participants of a 9-month aerobic and resistance exercise program performed three times a week during hemodialysis sessions had improved PDF, depression, cognitive function, and chronic fatigue at the end of the study period.^96^ In a RCT, hemodialysis patients assigned to intradialytic exercise two times a week for 36 weeks had a significant difference in general fatigue scores at 24 and 36 weeks when compared with controls.^97^ Intradialytic aerobic exercise for as little as 15 minutes/session for 8 weeks was shown to improve fatigue in chronic hemodialysis.^98^ Alternatively, less structured physical activity, *e.g*., increasing step counts, may be more favorable for some patients. Studies demonstrated that physical activity/step counts were negatively associated with PDF^99^ and chronic fatigue in MHD.^100^ Importantly, individual patient abilities and preferences must be considered when adopting exercise and/or physical activity as PDS interventions.

### CBT

There is evidence supporting the use of CBT for management of PDS symptoms.^101,107^ In a RCT in patients on MHD, a technology-assisted stepped collaborative care intervention involving 12 weekly CBT sessions±pharmacotherapy resulted in a significant decrease in general fatigue and pain severity at 3 and 6 months postintervention and a small, significant reduction in depression at 3 months.^102^ In a RCT in patients on chronic hemodialysis experiencing fatigue, there were moderate to large effects in favor of a CBT intervention (versus control) for fatigue severity, fatigue-related functional impairment, depression, and anxiety.^103^ Qualitative interviews from this RCT revealed that acceptability and engagement were facilitated by coherence between patients' understanding of fatigue and the CBT-based treatment but were limited by existing illness/treatment burden and the intervention's cognitive burden.^108^ This suggests that thoughtful implementation strategies must be used to make CBT interventions appealing, practical, and feasible options for the dialysis population. These investigations benefit from their design as multicenter RCTs but are limited by moderate to small sample sizes and studying only certain PDS dimensions.

### L-Carnitine

Carnitine is a cofactor that is essential for energy metabolism and prevention of organic acid accumulation in the mitochondria.^109^ Sources of carnitine include dietary intake and synthesis in the kidney and liver.^109^ Maintenance dialysis may result in carnitine depletion through dialytic membrane losses and decreased renal synthesis, which can exacerbate intradialytic symptoms and ESKD complications.^110^ A systematic review examining L-carnitine supplementation in dialysis patients found it may have little or no effect on fatigue, muscle weakness, SF-36 physical component scores, and QOL, but it may improve SF-36 mental component scores.^110^

## Conclusions

PDS is a pervasive and debilitating cluster of symptoms that is often under-recognized and undertreated. It is characterized by heterogeneous symptoms that are exacerbated after dialysis, including physical, cognitive, psychological, and behavioral disturbances and impairments. Our findings suggest that PDS has profound effects on QOL and daily functioning, yet there are no established methods or guidelines to define, diagnose, and manage it. In the interim, a pragmatic approach given the lack of evidence to address PDS in dialysis patients is recommended. First, we suggest collecting a detailed medical history from the patient to understand the range, severity, and effect of postdialysis symptoms. Next, consider using available PROMs to quantify symptoms and treatment effects. Finally, employ shared decision-making with patients when selecting among interventions, *e.g*., adjusting dialysis prescription, exercise and physical activity, and CBT, among others. In research settings, we propose a contemporary approach to PDS involving semistructured interviews^8^ and focus/nominal groups^75^ to better understand patients' views on its scope and extent of impact. This will guide future patient-centered studies and translation of research findings into clinical practice.

Further research should aim to establish a case definition and diagnostic criteria for PDS. In parallel, additional investigations are needed to develop measurement instruments that assess the breadth of PDS symptoms. This work will require qualitative methods to capture the voice of patients, families, and clinicians. Research on management strategies should be informed by RCTs on patients with PDS, using both dialysis and nondialysis interventions, and will benefit from interdisciplinary collaboration for effective clinical implementation.

## Supplementary Material
