# Supplementary material for: Post-Dialysis Syndrome: A Narrative Review
Source: Kidney360. 2025 Nov 10;7(5):1168–84. doi: 10.34067/KID.0000001062 (PMC13229444; doi:10.34067/KID.0000001062)
Supplement: Supplementary file 1 [file kidney360-7-1168-s001.pdf]

ASN American Society of Nephrology | JASN Journal of the American Society of Nephrology | KIDNEY360 Accessing Our World From Every Angle | CJASN Clinical Journal of the American Society of Nephrology

ASN Journal Disclosure Form

As per ASN journal policy, I have disclosed any financial relationships or commitments I have held in the past 36 months as included below. I have listed my Current Employer below to indicate there is a relationship requiring disclosure. If no relationship exists, my Current Employer is not listed.

G. Chacon-Palma reports the following:
Employer: University of New Mexico School of Medicine; and Research Funding: Dialysis Clinic, Inc.

I understand that the information above will be published within the journal article, if accepted, and that failure to comply and/or to accurately and completely report the potential financial conflicts of interest could lead to the following: 1) Prior to publication, article rejection, or 2) Post-publication, sanctions ranging from, but not limited to, issuing a correction, reporting the inaccurate information to the authors' institution, banning authors from submitting work to ASN journals for varying lengths of time, and/or retraction of the published work.

Name: Gabriela Chacon-Palma
Manuscript ID: K360-2025-000774R1
Manuscript Title: Post-Dialysis Syndrome: A Narrative Review
Date of Completion: September 13, 2025
Disclosure Updated Date: September 13, 2025

ASN Journal Disclosure Form

As per ASN journal policy, I have disclosed any financial relationships or commitments I have held in the past 36 months as included below. I have listed my Current Employer below to indicate there is a relationship requiring disclosure. If no relationship exists, my Current Employer is not listed.

S. Erickson reports the following:
Employer: UNM

I understand that the information above will be published within the journal article, if accepted, and that failure to comply and/or to accurately and completely report the potential financial conflicts of interest could lead to the following: 1) Prior to publication, article rejection, or 2) Post-publication, sanctions ranging from, but not limited to, issuing a correction, reporting the inaccurate information to the authors' institution, banning authors from submitting work to ASN journals for varying lengths of time, and/or retraction of the published work.

Name: Sarah Jane Erickson
Manuscript ID: K360-2025-000774R1
Manuscript Title: Post-Dialysis Syndrome: A Narrative Review
Date of Completion: September 12, 2025
Disclosure Updated Date: September 12, 2025

ASN
American Society of Nephrology
JASN
Journal of the American Society of Nephrology
KIDNEY360
Accessing Our World From Every Angle
CJASN
Clinical Journal of the American Society of Nephrology

ASN Journal Disclosure Form

As per ASN journal policy, I have disclosed any financial relationships or commitments I have held in the past 36 months as included below. I have listed my Current Employer below to indicate there is a relationship requiring disclosure. If no relationship exists, my Current Employer is not listed.

M. Jhamb reports the following:
Employer: University of Pittsburgh and University of Pittsburgh Medical Center; Consultancy: Boehringer Ingelheim LLC, CKD Networks of Excellence, Eli-Lilly, Clinical Care Targeted Communications group, LLC; Research Funding: NIH, CKD Leaders Network of Excellence; and Other Interests or Relationships: Member of ASN, AHA, AMIA and National Kidney Foundation.

I understand that the information above will be published within the journal article, if accepted, and that failure to comply and/or to accurately and completely report the potential financial conflicts of interest could lead to the following: 1) Prior to publication, article rejection, or 2) Post-publication, sanctions ranging from, but not limited to, issuing a correction, reporting the inaccurate information to the authors' institution, banning authors from submitting work to ASN journals for varying lengths of time, and/or retraction of the published work.

Name: Manisha Jhamb
Manuscript ID: K360-2025-000774R1
Manuscript Title: Post-Dialysis Syndrome: A Narrative Review
Date of Completion: September 12, 2025
Disclosure Updated Date: April 8, 2025

ASN
American Society of Nephrology
JASN
Journal of the American Society of Nephrology
KIDNEY360
Accessing Our World From Every Angle
CJASN
Clinical Journal of the American Society of Nephrology

ASN Journal Disclosure Form

As per ASN journal policy, I have disclosed any financial relationships or commitments I have held in the past 36 months as included below. I have listed my Current Employer below to indicate there is a relationship requiring disclosure. If no relationship exists, my Current Employer is not listed.

M. Roumelioti reports the following:
Employer: University of New Mexico; Consultancy: My spouse: Quanta; Advisory or Leadership Role: Chair of the Medical Board ESRD Network 13; and Other Interests or Relationships: Participating in DCI quality meetings and receiving financial support.

I understand that the information above will be published within the journal article, if accepted, and that failure to comply and/or to accurately and completely report the potential financial conflicts of interest could lead to the following: 1) Prior to publication, article rejection, or 2) Post-publication, sanctions ranging from, but not limited to, issuing a correction, reporting the inaccurate information to the authors' institution, banning authors from submitting work to ASN journals for varying lengths of time, and/or retraction of the published work.

Name: Maria-Eleni Roumelioti
Manuscript ID: K360-2025-000774R1
Manuscript Title: Post-Dialysis Syndrome: A Narrative Review
Date of Completion: September 12, 2025
Disclosure Updated Date: March 11, 2025

ASN
American Society of Nephrology
JASN
Journal of the American Society of Nephrology
KIDNEY360
Accessing Our World From Every Angle
CJASN
Clinical Journal of the American Society of Nephrology

ASN Journal Disclosure Form

As per ASN journal policy, I have disclosed any financial relationships or commitments I have held in the past 36 months as included below. I have listed my Current Employer below to indicate there is a relationship requiring disclosure. If no relationship exists, my Current Employer is not listed.

J. Steel reports the following:
Employer: University of Pittsburgh; Consultancy: Juxtapose; and Patents or Royalties: Springer.

I understand that the information above will be published within the journal article, if accepted, and that failure to comply and/or to accurately and completely report the potential financial conflicts of interest could lead to the following: 1) Prior to publication, article rejection, or 2) Post-publication, sanctions ranging from, but not limited to, issuing a correction, reporting the inaccurate information to the authors' institution, banning authors from submitting work to ASN journals for varying lengths of time, and/or retraction of the published work.

Name: Jennifer L. Steel
Manuscript ID: K360-2025-000774R1
Manuscript Title: Post-Dialysis Syndrome: A Narrative Review
Date of Completion: September 12, 2025
Disclosure Updated Date: September 12, 2025

ASN
American Society of Nephrology
JASN
Journal of the American Society of Nephrology
KIDNEY360
Accessing Our World From Every Angle
CJASN
Clinical Journal of the American Society of Nephrology

ASN Journal Disclosure Form

As per ASN journal policy, I have disclosed any financial relationships or commitments I have held in the past 36 months as included below. I have listed my Current Employer below to indicate there is a relationship requiring disclosure. If no relationship exists, my Current Employer is not listed.

M. Unruh reports the following:
Employer: University of New Mexico; New Mexico Veterans Hospital; Research Funding: Dialysis Clinic Inc. for investigator initiated research.   I take no salary.; and Other Interests or Relationships: VOICE DSMB US Renal.

I understand that the information above will be published within the journal article, if accepted, and that failure to comply and/or to accurately and completely report the potential financial conflicts of interest could lead to the following: 1) Prior to publication, article rejection, or 2) Post-publication, sanctions ranging from, but not limited to, issuing a correction, reporting the inaccurate information to the authors' institution, banning authors from submitting work to ASN journals for varying lengths of time, and/or retraction of the published work.

Name: Mark L. Unruh
Manuscript ID: K360-2025-000774R1
Manuscript Title: Post-Dialysis Syndrome: A Narrative Review
Date of Completion: September 13, 2025
Disclosure Updated Date: September 13, 2025
